# Detection and Analysis of Human Cells Based on Artificial Neural Network

**DOI:** 10.1155/2022/4600840

**Published:** 2022-08-31

**Authors:** Jun Yao, Hongji Yuan

**Affiliations:** College of Computer Information and Engineering, Nanchang Institute of Technology, Nanchang 330044, China

## Abstract

The detection and classification of histopathological cell images is a hot topic in current research. Medical images are an important research direction and are widely used in computer-aided diagnosis, biological research, and other fields. A neural network model based on deep learning is also common in medical image analysis and automatic detection and classification of tissue and cell images. Current medical cell detection methods generally do not consider that the yield is affected by other factors in the topological region, which leads to inevitable errors in the accuracy and generalization of the algorithm; at the same time, the current medical cell imaging methods are too simple to predict the classification markers, which affect the accuracy of cell image classification. This study introduces the concepts of two kinds of neural networks and then constructs a cell recognition model based on the convolution neural network principle and staining principle. In the experimental part, we developed three groups of experiments using the same equation as the experiment and tested the best cell recognition model proposed in this study.

## 1. Introduction

In this study, we propose a study to train neural network simulators using biosphere flux data collected by EUROFLUX project to provide spatial and temporal estimates of European forest carbon flux on the continental scale. The novelty of this method is that the neural network structure is constrained and parameterized using traffic data, and a limited number of input driving variables are used [1]. In this study, a hybrid intelligent system based on past financial performance data is proposed, which combines a rough set method with neural network to predict enterprise failure. By comparing the traditional discriminant analysis and neural network method with our hybrid method, the effectiveness of this method is verified [2]. The artificial neural network method is used to predict short-term load of large-scale energy system. Different neuron combinations were used to test networks with one or two potential layers, and the prediction errors of the results were compared. When the neural network is divided into different load patterns, it can give a good load forecast [3]. The improved criteria of WG and MPA are established and verified using the artificial neural network and traditional methods. A multicenter study was conducted on 240 WG patients and 78 MPA patients. Appropriately trained neural networks and CT can distinguish these diseases and perform better than LR [4]. The support vector machine (SVM) and artificial neural network (ANN) systems are applied to the drug/non-drug classification problem as an example of the binary decision-making problem in the early virtual compound filtering. The results show that compared with the artificial neural network, the solution obtained by support vector machine training has better robustness and smaller standard error [5]. In this study, a new method based on the artificial neural network is proposed to identify MHCII binding cores and binding affinities simultaneously. A new training algorithm is used for training, which allows the correction of deviations in training data caused by redundant binding kernel representations [6]. This study introduces the implementation of FANN, which is a fast artificial neural network library written by ANSIC. The results show that the speed of FAAN library is obviously faster than other libraries on the system without floating-point processor, while the performance of FANN library on the system with floating-point processor is equivalent to other highly optimized libraries [7]. The purpose of this study was to determine whether circulating tumor cells were present in the blood of patients with large operable or locally advanced breast cancer before and after neoadjuvant chemotherapy and before and after preoperative neoadjuvant chemotherapy. After research, we concluded that in patients receiving neoadjuvant chemotherapy, CELLSEARCH system can detect circulating tumor cells in a low truncation range of 1 cell. Detection of circulating tumor cells is not associated with primary tumor response, but is an independent prognostic factor for early recurrence [8]. The pathological TNM stage is the best factor to judge the prognosis of non-small cell lung cancer. After isolating NSCLC patients by the size of epithelial tumor cells, cytological analysis was used to evaluate the presence of CTC in surgical patients [9]. In this study, a microbial electronic manipulation and detection lab-on-a-chip based on a closed dielectrophoresis cage combined with impedance sensing is proposed. This method is suitable for implementation in integrated circuit technology, which can not only operate and detect a single unit but also reduce the scale of the system [10]. Circulating tumor cells have long been considered to reflect the invasiveness of tumors. Therefore, many people have tried to develop analytical methods to reliably detect and enumerate CTCs, but such analytical methods have not been available until recently. This article reviews CTCs, especially the technical problems of its detection, the clinical results obtained so far, and the future prospects [11]. To determine the clinical application of immunoglobulin heavy chain gene rearrangement identification in multiple myeloma tumor cell detection, we investigated 36 consecutive newly diagnosed patients intending to receive high-dose chemotherapy in a research program. There is no consistent relationship between bone marrow MRD status and clinical course, and patients with negative PCR also have early recurrence [12]. Using yeast cells as a model system, a piezoelectric lead zirconate titanate-stainless steel cantilever beam was studied as a real-time cell detector in water. Under the experimental conditions, when the cell diffusion distance is less than the linear size of the adsorption area, the resonance frequency shift rate has a linear relationship with the cell concentration, and the resonance frequency shift rate can be used to quantify the cell concentration [13]. Although optical cell counting and flow cytometry devices have been widely reported, there is usually a lack of sensitive and effective nonoptical methods to detect and quantify large surface area cells attached to micro-devices. We describe an electrical method based on measuring cell count changes in the conductivity of the surrounding medium due to ions released by immobilized cells on the inner surface of the microfluidic channel [14]. Background of the diagnostic value and prognostic significance of circulating tumor cell detection in bladder cancer are still controversial. We conducted a meta-analysis to consolidate the current evidence of using CTC detection methods to diagnose bladder and other urothelial cancers and the association between CTC-positive and advanced and remote diseases. Conclusion of CTC evaluation can confirm the diagnosis and differential diagnosis of bladder cancer [15].

## 2. Artificial Neural Network

### 2.1. RBF Neural Network

RBF neural network belongs to a kind of radial neural network. When there are enough nerve cells in the hidden layer, it can be designed as any continuous function infinitely. Local approximation, classification, and pattern recognition are all very good, and the learning and teaching time of the algorithm is very short. The mapping relation in RBF neural network is expressed as *f*(*x*) : *R*_*n*_⟶*R*_*o*_, as shown as follows:

(1) $$\begin{array}{c} y=f\left(x\right)=\sum_{i=1}^{c}\omega_{i}\varphi \left(\left\|x-c_{i}\right\|,\sigma_{i}\right)=\sum_{i=1}^{c}\omega_{i}\exp\left(-\frac{{\left\|x-c_{i}\right\|}^{2}}{2\sigma_{i}^{2}}\right), \end{array}$$

where *C* is the number of neurons in the potential layer of the network, *c*_*i*_ is the center of radial basis function of each potential layer, the width is *σ*_*i*_, and *ω*_*i*_ is the *i*th activation function and exit neuron. The neural network of RBF must be trained and learned to determine the radial basis center *c*_*i*_, width *σ*_*i*_, and weight *ω*_*i*_ between the potential layer and the output layer of neurons in each potential layer, to determine the mapping relationship between inputs.

To ensure that each activation function is not peaceful or too sharp, the activation function of latent neurons is regarded as a fixed radial basis function, and the center *c*_*i*_ of latent radial basis function is randomly selected from training. The radial basis function is defined as follows:

(2) $$\begin{array}{c} \varphi \left(x,c_{i}\right)=\exp\left(-\frac{k}{d_{\max}}{\left\|x-c_{i}\right\|}^{2}\right), \end{array}$$

where *K* represents the number of neurons in the hidden layer and *d*_max_ is the maximum distance between the two centers, and this formula shows that the width of neurons in the hidden layer is constant.

### 2.2. BP Neural Network

**Figure 1:**
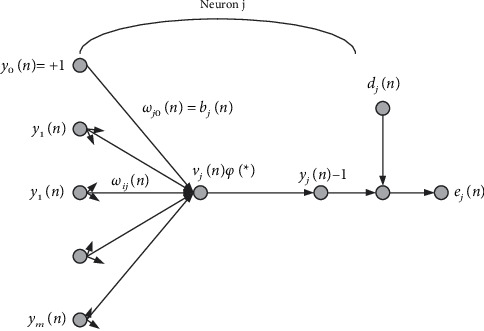
Schematic diagram of BP neural network reverse algorithm.

BP neural network is a multilayer feedback neural network with inverse error transmission. The learning process can be divided into signal transmission and error reverse transmission. A schematic diagram of BP neural network reverse transmission algorithm is shown in Figure 1.

From this, it can be deduced that the weight correction values are shown as follows:

(3) $$\begin{array}{c} \Delta \omega_{ji}\left(n\right)=\eta \times \delta_{j}\left(n\right)y_{i}\left(n\right), \end{array}$$

(4) $$\begin{array}{c} \delta_{j}\left(n\right)=\left(d_{j}\left(n\right)-y_{j}\left(n\right)\right)\varphi_{j}\left(\sum_{i=0}^{m}\omega_{ji}\left(n\right)y_{i}\left(n\right)\right), \end{array}$$

where *M* represents all the inputs that affect neuron *J*, *η* is the inverse error rate of learning, *δ*_*j*_(*n*) is the local gradient, *y*_*i*_(*n*) is the output of neuron *I*, and Ψ is the activation function.

## 3. Research on Cell Image Detection

### 3.1. Construction of Cell Image Detection Network Model

#### 3.1.1. Principle of Convolution Neural Network

The convolution neural network is based on the mathematical mapping in this study. It can learn the same mapping ability as this expression independently. It specializes in learning that needs to be practiced in a specific space, so this training can make it learn the mapping relationship between input and output. The process is shown as follows:

(5) $$\begin{array}{c} y=g\left(x;w_{1},\cdots ,w_{L}\right),\\ =g_{L}\left(\cdot ;w_{L}\right)\circ g_{L-1}\left(\cdot ;w_{L-1}\right)\circ \cdots \circ g_{2}\left(\cdot ;w_{2}\right)\circ g_{1}\left(x;w_{1}\right), \end{array}$$

where *y* represents output vector, *x* represents input vector, *g* represents CNN, *g*_*L*_ represents layer 1 CNN, *w*_*L*_ represents layer 1 *g*_*L*_ weight and bias vector, and ° represents convolution operation.

**Figure 2:**
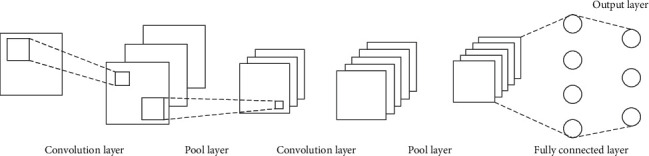
Basic structure diagram of convolution neural network.

A convolution neural network is usually composed of the following types (as shown in Figure 2). The convolution layer is used to separate important functions, the pooling layer is used to reduce the number of parameters and excessive matching, and the complete combination layer is usually used for network output after all convolution operations.

Input Layer: This layer is used to input data. In multidimensional data processing, because the input data are usually images, this study mainly introduces the input layer of objects placed in images. First, the image information is converted into function data and input into convolution neural network. The image structure is the embodiment of image information. In analysis, the CNN input layer keeps its original data when processing image information. Images are usually divided into black and white images and color images. When CNN analyzes different types of images, the inputs are different.

Convolution Layer: the convolution layer first detects each feature of the image locally and then performs local expansion processing at a higher level to obtain global information. The core of convolution operation is a mathematical operation, which usually represents discrete convolution in convolution neural network. The convolution formula is as follows:

(6) $$\begin{array}{c} x_{i}^{l}=f\left(\sum_{i}w_{i,j}^{l-1,l}\circ x_{i}^{l-1}+b_{j}^{l}\right), \end{array}$$

where *x*_*i*_^*l*^ represents the *i*-level image of the *i* level, *x*_*i*_^*l*−1^ represents the first to *i*-level images, ∘ is the convolution operator, *w*_*i*,*j*_^*l*−1,*l*^ is the first to *i*-level images and *l* − 1, *b*_*j*_^*l*^ represents the offset of the *j*th feature corresponding to the *l* level, and *F* represents the activation function. The most common of these activation functions is the relay-type activation function, whose principle is shown as follows:

(7) Relu=max0,x=x,x≥0,0,x<0.

Pooling Layer: pooling layer is usually combined with the convolution layer, which is mainly used to reduce function scale, compare data, reduce the number of network parameters, reduce overmatching, and improve the tolerance of fault model. Complete Combination Layer: after processing several convolution layers and a pooling layer, the convolution neural network will be combined with the complete combination layer. Output Layer: the focus of the output layer of convolution neural network is to produce the desired results according to the situation. After calculation, different probability values are obtained from input to output.

#### 3.1.2. Proposition and Construction of Cell Image Detection Model

Assuming that the time domain remains constant, Ω is defined as the state region of output *Y*, which is based on the finite state of the model. Suppose that the spatial constrained regression model *g* is used to test the known and has *y*=*g*(Ω; *s*(*x*)) form, where *s*(*x*) is an unknown parameter vector, and the result of the last layer of ordinary CNN is shown as follows:

(8) $$\begin{array}{c} y=f_{L}\left(x_{L-1};w_{L}\right), \end{array}$$

where *x*_*L*−1_ is the output of the network (*L* − 1) layer in the neural network and *w*_*L*_ is the weight of the last layer, which is output under the mapping of *f*_*L*_. Based on the theoretical analysis of space constraints in this study, we need to extend the standard CNN to estimate *s*(*x*) so that the last two layers (*f*_*L*−1_, *f*_*L*_) of the network are defined as follows:

(9) $$\begin{array}{c} s\left(x\right)=f_{L-1}\left(x_{L-2};w_{L-1}\right), \end{array}$$

(10) $$\begin{array}{c} y=f_{L}\left(\Omega ;s\left(x\right)\right)=g\left(\Omega ;s\left(x\right)\right), \end{array}$$

where *x*_*L*−2_ is the output of the network (*L* − 2) layer and Formula (9) is the parameter estimation layer. According to the weight *w*_*L*−1_, printing the image to obtain a parameter vector; Formula (10) is the spatial constraint layer, which belongs to the parameter vector in the regression model.

At the beginning of kernel image recognition, image plane *x* ∈ *R*^*H*×*W*×*D*^, height *H*, width *W,* and feature number *D* are given, and the goal is to detect the center point *X* of each kernel.

In this study, the Euclidean distance from the pixel to the core, i.e., ‖*Z*_*j*_ − *Z*_*m*_^0^‖_2_, is obtained when the core is detected, where *Z*_*j*_ and *Z*_*m*_^0^ represent the coordinates of *y*_*j*_ and the center coordinates of the *m*th core, respectively. The weight is reduced, i.e., normalized, and the regularized formula is shown as follows:

(11) $$\begin{array}{c} d=\frac{1}{2}{\left\|Z_{j}-Z_{m}^{0}\right\|}_{2}^{2}. \end{array}$$

Let Ω = {1, ⋯, *H*′}*∗*{1, ⋯, *w*′}, and y is the spatial region. The j-th element is *j* = 1,…, |Ω|. Equation (12) is defined as follows:

(12) $$\begin{array}{c} y_{j}=\left\{\frac{1}{1+\left({\left\|Z_{j}-Z_{m}^{0}\right\|}_{2}^{2}/0^{2}\right)}\right.\underset{\text{otherwise},}{\text{if}\forall m\neq m\prime ,{\left\|Z_{j}-Z_{m}^{0}\right\|}_{2}\leq {\left\|Z_{j}-Z_{m\prime }^{0}\right\|}_{2}\leq d}, \end{array}$$

where *Z*_*j*_ and *Z*_*m*_^0^ represent the coordinates of *y*_*j*_ and the center coordinate of the *m*th core of *D*, respectively, and Ω is a constant radius. It can be seen from the figure that the probability graph defined by Equation (12) has a maximum value near the center of each core *Z*_*m*_^0^, and other places are flat. Next, a prediction output $\hat{y}$ generated from a space-constrained layer of the network is determined. Based on the known structure of the motion result probability graph described in Equation (12), we define the predicted output as Equation (13) of the *J*th element.

(13) $$\begin{array}{c} {\hat{y}}_{j}=g\left(Z_{j};{\hat{Z}}_{1}^{0},\cdots ,{\hat{Z}}_{M}^{0},h_{1},\cdots ,h_{M}\right),\\ =\left\{\frac{1}{1+\left({\left\|Z_{j}-Z_{m}^{0}\right\|}_{2}^{2}/0^{2}\right)}\right.\underset{\text{otherwise},}{\text{if}\forall m\neq m^{\prime },{\left\|Z_{j}-{\hat{Z}}_{m}^{0}\right\|}_{2}\leq {\left\|Z_{j}-{\hat{Z}}_{m\prime }^{0}\right\|}_{2}\leq d}, \end{array}$$

where ${\hat{Z}}_{m}^{0}\in \Omega$ represents the center of the formula estimate, *h*_*M*_ ∈ [0,1] represents the height of the *m*th variable, and *M* represents the maximum number on $\hat{y}$. Because of the redundancy provided by *h*_*M*_ = 0 or ${\hat{Z}}_{m}^{0}={\hat{Z}}_{m}^{0},m\neq m^{\prime }$, $\hat{y}$ defined in this way will occur to allow the number of prediction cores to change from 0 to *M*. In the experiment, *D* in Formula (12) and Formula (13) is set to 4 pixels to provide sufficient support area for the probability mask.

Parameters ${\hat{Z}}_{m}^{0}=\left(u_{m},v_{m}\right)$ and *h*_*m*_ are estimated in a parameter estimation layer. *X*_*L*−2_ is made the output of the (*L* *−* 2) layer of the network. *u*_*m*_, *v*_*m*_, *h*_*m*_ are defined as follows:

(14) $$\begin{array}{c} u_{m}=\left(H^{\prime }-1\right)*\text{sigm}\left(W_{L-1,u_{m}}*X_{L-2}+b_{u_{m}}\right)+1, \end{array}$$

(15) $$\begin{array}{c} v_{m}=\left(W^{\prime }-1\right)*\text{sigm}\left(W_{L-1,v_{m}}*X_{L-2}+b_{v_{m}}\right)+1, \end{array}$$

(16) $$\begin{array}{c} h_{m}=\text{sigm}\left(W_{L-1,h_{m}}*X_{L-2}+b_{h_{m}}\right). \end{array}$$

The purpose of formulas (14) and (15) is to show that the corresponding weights and deviations are output to the previous layer, then normalized, and then combined with the previous predictions to obtain a parameter estimate.

The importance of Formula (16) is that it is useful for the upper exit. After the corresponding weights and deviations are given, normalization is carried out to obtain the estimated height of *M* variable, which fully integrates the spatial area position data. When *b*_*u*_*m*__, *b*_*v*_*m*__, *b*_*h*_*m*__ and *W*_*L*−1,*u*_*m*__, *W*_*L*−1,*v*_*m*__, *W*_*L*−1,*h*_*m*__ are vectors, the former represents deviation, the latter represents weighting, and sigm(·) represents the sigmoid function commonly used in convolution neural networks, which is often used to hide the output of neurons, and its value range is (0, 1). It can specify a real number between (0, 1); that is, it is used for normalization. The principle is shown as follows:

(17) $$\begin{array}{c} S\left(x\right)=\frac{1}{1+e^{x}}, \end{array}$$

where *X* represents the data after zero mean processing and *S*(*x*) represents the data after normalization processing, and the learning method should use a loss function, as shown as follows:

(18) $$\begin{array}{c} l\left(y,\hat{y}\right)=\sum \left(y_{j}+\varepsilon \right)H\left(y_{j},{\hat{y}}_{j}\right), \end{array}$$

where *ε* is a small constant, which represents the ratio of nonzero probability pixels to the total number of zero probability pixels in the training input, and $H\left(y_{j},{\hat{y}}_{j}\right)$ is the cross-entropy loss, which is specifically defined as follows:

(19) $$\begin{array}{c} H\left(y_{j},{\hat{y}}_{j}\right)=-\left[y_{j}\log\left({\hat{y}}_{j}\right)-\left(1-y_{j}\right)\log\left(1-{\hat{y}}_{j}\right)\right]. \end{array}$$

Among them, when the actual values are *y*_*j*_=1 and $H\left(y_{j},{\hat{y}}_{j}\right)=-\log\left({\hat{y}}_{j}\right)$, when the predicted value of ${\hat{y}}_{j}$ is closer to 1, $\log\left({\hat{y}}_{j}\right)$ is closer to the maximum value of 1, and the minus sign indicates the minimum error value. When the predicted value of ${\hat{y}}_{j}$ is closer to zero, $\log\left({\hat{y}}_{j}\right)$ is closer to the negative. An infinite addition and subtraction sign indicates the maximum error value. When the actual values are *y*_*j*_=0 and $H\left(y_{j},{\hat{y}}_{j}\right)=-\log\left(1-{\hat{y}}_{j}\right)$, when the predicted value ${\hat{y}}_{j}$ is closer to zero, $\log\left({\hat{y}}_{j}\right)$ is closer to the maximum value 1, and the minus sign indicates the minimum error value, while when the predicted value ${\hat{y}}_{j}$ is closer to 1, $\log\left({\hat{y}}_{j}\right)$ is closer to the negative infinite addition and subtraction sign, which represents the maximum error value.

The detailed parameters of each convolution are shown in Table 1.

In Table 1, you can see that the input is an input attribute with a size of 27 × 27, and the output attribute after the final network frame is 11 × 11. To extract and merge all function information, the scroll window increment is always set to 1, and the trigger function uses relay-type trigger function evenly.

**Figure 3:**
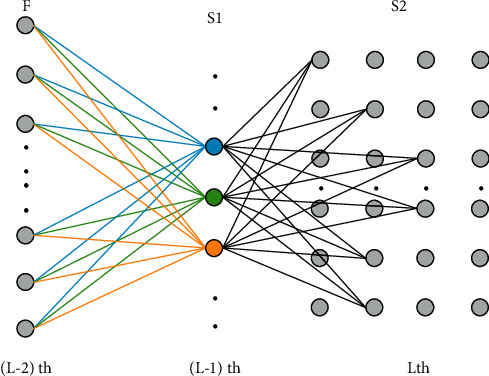
Structure of cell detection model.

The network model structure mentioned in this article is shown in Figure 3.

*F* is the full interconnection layer, and these neurons in the full interconnection layer represent medical image information without spatial information; S1 is a new parameter estimation layer, and these neurons in the parameter estimation layer represent the estimated position information; S2 is the spatial constraint layer, *L* is the total number of layers in the network, and each neuron represents the medical image information with state parameter information.

### 3.2. Nuclear Image Preprocessing

#### 3.2.1. Coloring Principle of Stain

The color deconvolution method is mainly based on the orthogonal transformation of the original RGB image, and according to the Beer–Lambert law, it is expressed as the relationship between the light intensity of the histological cell image and the staining matrix, as shown as follows:

(20) $$\begin{array}{c} I_{C}=I_{O,C}\exp\left(-Q*C_{C}\right), \end{array}$$

where *I*_*O*,*C*_ is the intensity of incident light radiated from the tissue cell image, *I*_*C*_ is the intensity of light passing through the tissue cell image, subscript *C* is the RGB three-channel identifier, *Q* is the dye color matrix, and *C* is the dye absorbance. It can be seen from Equation (10) that the intensity of transmitted light and dye content is relatively complex nonlinear relations. In the RGB color model, the light intensity of each pixel in the camera is *I*_*R*_, *I*_*G*_, and *I*_*B*_, respectively. The optical density (OD) expression of each pixel is shown as follows:

(21) $$\begin{array}{c} {\text{OD}}_{C}=-\;\log_{10}\left(\frac{I_{C}}{I_{O,C}}\right)=Q*C_{C}. \end{array}$$

It can be seen from Equation (21) that the optical density of each channel has a linear relationship with the absorption of light absorbent, so the optical density of each channel can be used to distinguish the color rendering effect of several dyes. The color effect of each point can be quantified by a 3 × 1 RGB three-channel optical density matrix. Using simple hematoxylin staining, the absorbance of R, G, and B channels was 0.18, 0.20, and 0.18, respectively. The size of the color matrix *Q* is related to the type of point, and each element of the matrix is proportional to the absorbance of each channel. For the three dyes R, G, B, the three-channel color system is defined as follows:

(22) $$\begin{array}{c} \left[\begin{array}{ccc} Y_{11} & Y_{12} & Y_{13}\\ Y_{21} & Y_{22} & Y_{23}\\ Y_{31} & Y_{32} & Y_{33} \end{array}\right]. \end{array}$$

Each row represents a dot, and each column represents the absorbance values of R, G, and B channels. In this data set, only two dyes are used for staining, and the corresponding chromosome systems of R, G, and B channels are shown as follows:

(23) $$\begin{array}{c} \left[\begin{array}{ccc} Y_{11} & Y_{12} & Y_{13}\\ Y_{21} & Y_{22} & Y_{23} \end{array}\right]. \end{array}$$

In the dyeing experiment, one dye was used to obtain the absorbance values of three RGB channels after dyeing with each dye. The dyeing formula for hematoxylin and eosin multiple dyeing is as follows:

(24) $$\begin{array}{c} \left[\begin{array}{ccc} 0.18 & 0.20 & 0.18\\ 0.01 & 0.13 & 0.01 \end{array}\right]. \end{array}$$

#### 3.2.2. Color Deconvolution

To make the color effect of each color in multicolor image stand out, RGB information must be transformed orthogonally. The purpose of orthogonal transformation is to make the color effect of each color independent of each other, to obtain the color effect of a dye. The transformed matrix must be normalized, and the normalization process for each dye is shown as follows:

(25) $$\begin{array}{c} {\hat{Y}}_{11}=\frac{Y_{11}}{\sqrt{Y_{11}^{2}+Y_{12}^{2}+Y_{13}^{2}}}, \end{array}$$

(26) $$\begin{array}{c} {\hat{Y}}_{21}=\frac{Y_{21}}{\sqrt{Y_{21}^{2}+Y_{22}^{2}+Y_{23}^{2}}}. \end{array}$$

The normalized optical density matrix *A* is shown as follows:

(27) $$\begin{array}{c} \left[\begin{array}{ccc} {\hat{Y}}_{11} & {\hat{Y}}_{12} & {\hat{Y}}_{13}\\ {\hat{Y}}_{21} & {\hat{Y}}_{22} & {\hat{Y}}_{23} \end{array}\right]. \end{array}$$

The *N* × 2 matrix *C* is used to describe the color effect of two dyes on a pixel, and then, the optical density matrix *Y* = *AC* of the image collected from the pixel is obtained. *C*=*A*^−1^  *Y*, and the color convolution matrix is then a pixel hint. Individual color effects can be determined according to the optical density and color moment of the image. The inverse of matrix *D*=*A*^−1^ is obtained.

The color deconvolution matrix of the above H&E coloring method is shown as follows:

(28) $$\begin{array}{c} \left[\begin{array}{ccc} 1.88 & -0.07 & -0.60\\ -1.02 & 1.13 & -0.48 \end{array}\right]. \end{array}$$

Multiple color images are separated by color deconvolution theory, and the separated images can be used for density and texture analysis.

**Figure 4:**
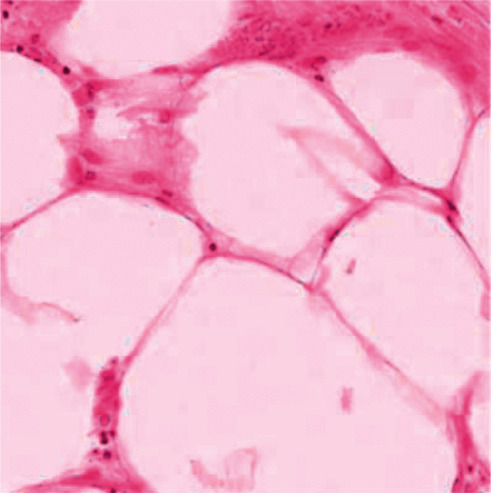
Sample image of H&E staining.

**Figure 5:**
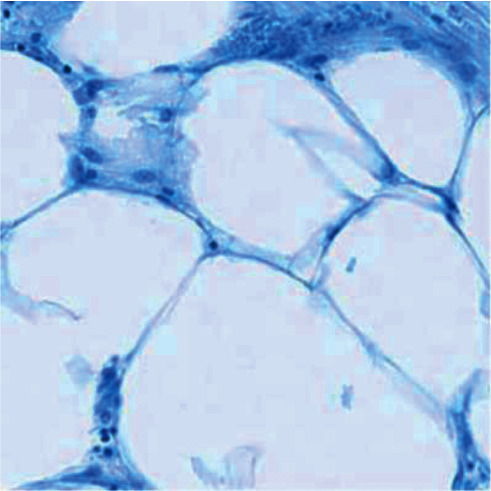
Images of hematoxylin staining components.

**Figure 6:**
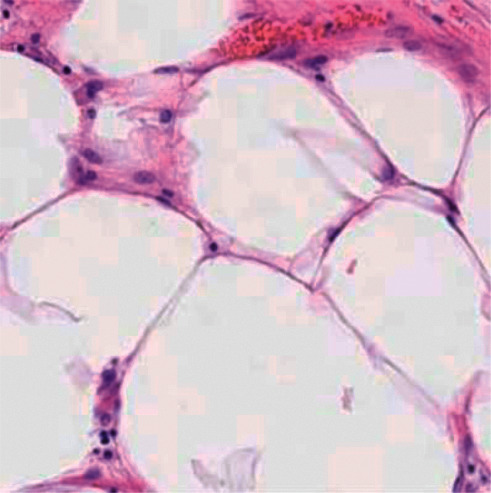
Eosin staining component image.

The cell sample images were experimented according to H&E staining mode, and the experimental results are shown in Figures 4–6.

In the image of the isolated hematoxylin-stained component, the nucleus is blue, while in the image of the eosin-stained component, the cytoplasm and cytoplasm are pink. After color inversion of the pathological picture of this material, the separation result of nucleus and cytoplasm is very good. As shown in the above figure, the color deconvolution method can be used as an image preprocessing method in this study.

## 4. Comparative Experiment and Analysis

### 4.1. Comparative Experiment and Analysis of Cell Detection

In this section, we designed the same control group as the experimental group, tested SCNN and SR-CNNSSAE models, respectively, and tested the parameters according to the detection performance of CRCStoPhenotypes data set.

This section selects 100 cell images from the test data set and stores the accuracy, recovery rate, and F1 scores of the three experimental models when testing the images. Tables 2–4 compare the differences in the three experimental systems in three evaluation indexes in detail.

Table 2 shows that the maximum recovery rate of SCNN is 0.9076, which is 0.0546 and 0.2146 higher than SR-CNN and SSAE, respectively, same but improved compared with 0.16 SSAE; on average, SCNN still leads SR-CNN and SSAE. SCNN through the maximum recovery rate, minimum recovery rate, and average recovery rate of comparative analysis shows that the detection accuracy has been greatly improved. However, the mean square error of SCNN is larger than that of SR-CNN and SSAE, which shows that the stability is not as good as that of SR-CNN and SSAE, but the difference is very small, only 0.01, which is within the range of acceptable area.

It can be seen from Table 3 that in terms of accuracy, the highest accuracy of SCNN is 0.8883, and SR-CNN and SSAE are 0.0503 and 0.2163, respectively; SCNN has a minimum accuracy of 0.7002. In SR-CNN and SSAE, the minimum values are 0.6801 and 0.5141, respectively; in terms of average accuracy, SCNN and SR-CNN are basically the same, only 0.002 behind, which belongs to the normal statistical range and is obviously ahead of SSAE. The above three groups of comparative data show that SCNN has an excellent performance in accuracy. In terms of stability, the three experimental systems are basically the same, and they are all relatively stable.

It can be seen from Table 4 that the maximum F1 of SCNN is 0.836947, which is 0.009 and 0.173 higher than SR-CNN and SSAE, respectively. Based on SR-CNN, the minimum F1 of SCNN is 0.70065. On the basis of SSAE, it decreased by 0.009 and increased by 0.132. For average, F1 of SCNN improved by 0.008 on SR-CNN but significantly surpassed SSAE. The above three sets of comparative data show that SCNN performed very well at F1. In terms of stability, the F1 score of SCNN in SR-CNN system is less than 0.005 and that in SSAE system is 0.001, which shows that this system is more stable.

The above analysis compares the detection performance differences in multiple cell images in detail from three indexes.  Table 5 analyzes and compares the total indexes of the three experimental systems.

Table 5 shows that SCNN has better performance than SR-CNN and SSAE in terms of recovery rate and F1 score. Although it lags behind SR-CNN in accuracy, the difference is very small, which indicates that the experimental performance can be relied on.

Summarizing the above experimental results and comparative analysis, this section shows that the SCNN cell recognition model proposed in this study has better detection accuracy and stronger generalization ability, which shows that it is very important to add spatial information to the designed convolution neural network model.

### 4.2. Comparative Experiment and Analysis of Cell Classification

This section contains three sets of comparative experiments with the same experimental settings, which are designed to test the classification ability of the kernel classification model, the kernel classification model, and the kernel classifcation model proposed in this paper for the CRCHistoPhenotypes dataset. The parameters of the comparative test are the same as those of the classification test in Chapter 3.

**Figure 7:**
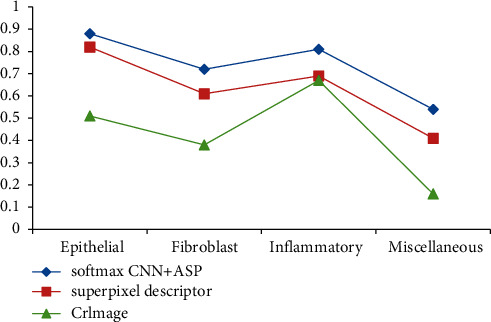
F1 scores were obtained by classifying different types of nuclei by three methods.

The F1 scores in different core classifications are compared, and the reference methods are CRImage method and superpixel imaging method. The exact F1 score is shown in Figure 7.

As can be seen from Figure 7, the F1 score of the classification method based on adjacent set prediction proposed in this study is higher than that of the other two methods in the four categories, and the curve is more stable, indicating the best performance. See Table 6 for a detailed comparison.

**Figure 8:**
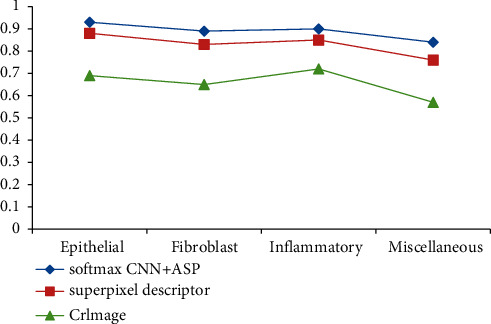
AUC values of four types of nuclear classification by three methods.

It can be seen from Table 6 that in terms of average F1, this method is obviously ahead of CRImage method in the super-pixel imaging method. The above three groups of comparative data show that the classification model based on adjacent set prediction in this study performs very well for F1 scores. In terms of stability, this method is 0.047 smaller than CRImage method, which shows that this model is more stable. In the same experimental environment, we combine SoftmaxCNN with a group of adjacent prediction methods, use CRImage method and superpixel imaging method to detect four different nuclei, and get the AUC values of different nuclei. See Figure 8 for details.

Figure 8 analyzes and compares the present model, the CRImage super-pixel imaging model, and the AUC metrics. Comparing these three curves, we can see that the model in this study has better AUC performance than the other two methods in the classification of four types of kernels.  Table 7 compares the differences in AUC statistical data of the three experimental schemes in detail.

As can be seen from Table 7, the AUC of prediction-based adjacent set classification model for four different core types is 0.059 and 0.217 higher than that of super-pixel imaging method and CRImage method, respectively, the minimum value is 0.099 and 0.295 higher, and the average value is higher, more than 0.071 and 0.2435. The performance of this model is better, and the mean square error is less than 0.0208 and 0.0346, which shows that the model in this study is more stable in classifying cell images. After comparing the F1 fraction and AUC values obtained from different types of nuclei, the weighted integration of F1 fraction and AUC values was carried out and a detailed comparison was made. The specific numerical equations are shown in Table 8.

Table 8 shows that the combination of SoftmaxCNN and AdjacentSetPrediction is used to classify the kernel used in this study, which is nearly 1 percentage point higher than the F1 score of the other two kernel classification methods, which is more. It shows the superiority of the proposed model in nuclear classification of cell histology image classification based on adjacent set prediction. The multi-class AUC is at least 0.6 percentage points higher than that of SuperpixelDescripto method and 2 percentage points higher than that of CRImage method. The combination of SoftmaxCNN and adjacent force prediction is more than 90% in multiclass. The comparison results of AUC values show that the proposed method has better classification ability and stability in nuclear classification.

Based on the above experimental results and comparative analysis, this section demonstrates that the proposed nuclear classification model based on adjacent set prediction has better classification ability and stronger stability and proves that convolution neural network combined with adjacent set prediction model is effective.

## 5. Concluding Remarks

In this study, we propose a method to detect nuclei by combining spatial data. This method aims at detecting nuclei in histological cell images and constructing a spatial model of cell image detection, to solve the problem of missing topological input in the current model. Aiming at the problem of how to classify the nuclei in the enlarged image of human cells, a prediction mechanism based on adjacent sets is proposed, and a large classification model of human cell images is constructed by combining the convolution neural network system of linear regression. In recent years, the deep learning method is widely used, which provides a theoretical basis for human cell image detection and classification combined with neural network model.

## Figures and Tables

**Table 1:** Design of parameter table of nuclear detection model based on spatial information.

Number of layers	Category	Filter size	Input/output dimensions
0	Input		27 × 27 × 1
1	conv1	4 × 4 × 1 × 36	24 × 24 × 36
2	pooling1	2 × 2	12 × 12 × 36
3	conv2	3 × 3 × 36 × 48	10 × 10 × 48
4	pooling2	2 × 2	5 × 5 × 48
5	Fully-connected1	5 × 5 × 48 × 512	1 × 512
6	Fully-connected2	1 × 1 × 512 × 512	1 × 512
7	sconv1	1 × 1 × 512 × 3	1 × 3
8	sconv2		11 × 11

**Table 2:** Quantitative table of detection and evaluation indexes.

	SCNN	SR-CNN	SSAE
Maximum value	0.9076	0.8531	0.6932
Minimum value	0.7011	0.7011	0.5411
Mean value	0.8234	0.8039	0.6439
Mean square error	0.0363	0.0264	0.0264

**Table 3:** Quantitative table of detection and evaluation indexes.

	SCNN	SR-CNN	SSAE
Maximum value	0.8823	0.8321	0.6662
Minimum value	0.7002	0.6801	0.5141
Mean value	0.7811	0.7829	0.6169
Mean square error	0.0285	0.0264	0.0264

**Table 4:** Quantitative table of detection and evaluation indexes.

	SCNN	SR-CNN	SSAE
Maximum value	0.8369	0.8276	0.6638
Minimum value	0.7006	0.7102	0.5688
Mean value	0.8007	0.7929	0.6296
Mean square error	0.0159	0.0208	0.0194

**Table 5:** Experimental comparison results.

Method	Precision	Recall	F1 score
SCNN	0.781	0.823	0.802
SR-CNN	0.782	0.804	0.793
SSAE	0.617	0.644	0.63

**Table 6:** Quantitative table of F1 fraction of different nuclear classifications by three methods.

	Softmax CNN + ASP	Super-pixel descriptor	CRImage
Maximum value	0.875	0.817	0.672
Minimum value	0.538	0.395	0.156
Mean value	0.7342	0.625	0.427
Mean square error	0.1692	0.177	0.216

**Table 7:** Quantification table of AUC values of four nuclear classifications by three methods.

	Softmax CNN + ASP	Super-pixel descriptor	CRImage
Maximum value	0.946	0.887	0.729
Minimum value	0.836	0.737	0.541
Mean value	0.901	0.831	0.657
Mean square error	0.047	0.068	0.081

**Table 8:** Comparison of nuclear classification results of three methods.

Method	F1 score	Multi-class AUC value
Softmax CNN + ASP	0.784	0.917
Super-pixel descriptor	0.687	0.853
CRImage	0.488	0.684

## Data Availability

The experimental data used to support the findings of this study are available from the corresponding author upon request.
